# [Al(H_2_O)_6_][Cr(OH)_6_Mo_6_O_18_]·10H_2_O

**DOI:** 10.1107/S1600536810053936

**Published:** 2011-01-12

**Authors:** Bao Li, Ling Ye, Li-Xin Wu

**Affiliations:** a State Key Laboratory of Supramolecular Structure and Materials, College of Chemistry, Jilin University, Changchun 130012, People’s Republic of China

## Abstract

The title compound, [Al(H_2_O)_6_][Cr(OH)_6_Mo_6_O_18_]·10H_2_O, hexa­aqua­aluminium hexa­hydroxidoocta­deca­oxido­molybdo­chromate(III) deca­hydrate, crystallizes isotypically with its gallium analogue [Ga(H_2_O)_6_][Cr(OH)_6_Mo_6_O_18_].10H_2_O. In the structure of the title compound, both the [Al(H_2_O)_6_]^3+^ cation and the Anderson-type [Cr(OH)_6_Mo_6_O_18_]^3−^ anion lie on centres of inversion. The anion is composed of seven edge-sharing octa­hedra, six of which are MoO_6_ octa­hedra that are arranged hexa­gonally around the central Cr(OH)_6_ octa­hedron. The anions are linked to each other by O—H⋯O hydrogen bonds into infinite chains along [100]. These chains are further connected with the [Al(H_2_O)_6_]^3+^ cations through O—H⋯O hydrogen bonds into sheets parallel to (01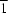). O—H⋯O hydrogen bonds involving all the lattice water mol­ecules finally link the sheets into a three-dimensional network.

## Related literature

For background literature on polyoxometalates, see: An *et al.* (2005 ▶); Shivaiah *et al.* (2003 ▶). The isotypic gallium analogue was reported by Kaziev *et al.* (2002 ▶).

## Experimental

### 

#### Crystal data

[Al(H_2_O)_6_][Cr(OH)_6_Mo_6_O_18_]·10H_2_O
*M* *_r_* = 1332.92
Triclinic, 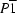

*a* = 6.809 (5) Å
*b* = 11.267 (7) Å
*c* = 11.596 (9) Å
α = 101.26 (3)°
β = 97.02 (3)°
γ = 101.81 (3)°
*V* = 841.7 (10) Å^3^
*Z* = 1
Mo *K*α radiation
μ = 2.63 mm^−1^
*T* = 290 K
0.12 × 0.12 × 0.11 mm

#### Data collection

Rigaku R-AXIS RAPID diffractometer
Absorption correction: multi-scan (*ABSCOR*; Higashi, 1995 ▶) *T*_min_ = 0.745, *T*_max_ = 0.770
8257 measured reflections
3801 independent reflections
3346 reflections with *I* > 2σ(*I*)
*R*_int_ = 0.037

#### Refinement

*R*[*F* ^2^ > 2σ(*F* ^2^)] = 0.033
*wR*(*F* ^2^) = 0.093
*S* = 1.10
3801 reflections
217 parameters
24 restraints
H-atom parameters constrained
Δρ_max_ = 0.89 e Å^−3^
Δρ_min_ = −0.86 e Å^−3^

### 

Data collection: *RAPID-AUTO* (Rigaku, 1998 ▶); cell refinement: *RAPID-AUTO*; data reduction: *CrystalStructure* (Rigaku/MSC, 2002 ▶); program(s) used to solve structure: *SHELXS97* (Sheldrick, 2008 ▶); program(s) used to refine structure: *SHELXL97* (Sheldrick, 2008 ▶); molecular graphics: *PLATON* (Spek, 2009 ▶); software used to prepare material for publication: *SHELXL97*.

## Supplementary Material

Crystal structure: contains datablocks global, I. DOI: 10.1107/S1600536810053936/wm2434sup1.cif

Structure factors: contains datablocks I. DOI: 10.1107/S1600536810053936/wm2434Isup2.hkl

Additional supplementary materials:  crystallographic information; 3D view; checkCIF report

## Figures and Tables

**Table 1:** Hydrogen-bond geometry (Å, °)

*D*—H⋯*A*	*D*—H	H⋯*A*	*D*⋯*A*	*D*—H⋯*A*
O*H*3—H3⋯O*T*1^i^	0.85	1.85	2.681 (4)	167
O*H*1—H1⋯O*B*2^i^	0.85	2.10	2.944 (4)	172
O*H*2—H2⋯O*W*5^ii^	0.85	1.82	2.665 (5)	178
O*W*3—H8⋯O*T*4^iii^	0.85	1.83	2.628 (5)	156
O*W*3—H9⋯O*W*7^iii^	0.85	1.81	2.632 (5)	163
O*W*1—H4⋯O*W*7	0.85	2.01	2.730 (5)	142
O*W*1—H5⋯O*W*4^iv^	0.85	1.74	2.577 (5)	167
O*W*2—H6⋯O*B*3^v^	0.85	1.72	2.559 (4)	171
O*W*2—H7⋯O*W*6	0.85	1.88	2.728 (5)	178
O*W*4—H10⋯O*B*1^vi^	0.85	1.94	2.737 (5)	156
O*W*5—H12⋯O*T*1^vii^	0.86	2.15	3.002 (6)	179
O*W*5—H13⋯O*W*6^viii^	0.85	1.99	2.842 (6)	180
O*W*6—H14⋯O*W*5^v^	0.85	2.04	2.800 (6)	149
O*W*6—H15⋯O*T*3^iii^	0.85	2.45	3.091 (6)	133
O*W*7—H16⋯O*T*3^iii^	0.85	2.13	2.878 (5)	146
O*W*7—H17⋯O*W*8	0.85	1.98	2.759 (5)	152
O*W*8—H18⋯O*T*1^ix^	0.85	2.19	2.902 (5)	141
O*W*8—H18⋯O*T*6^x^	0.85	2.62	3.241 (5)	131
O*W*8—H19⋯O*W*8^xi^	0.85	2.56	3.283 (6)	144

Symmetry codes: (i) 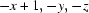; (ii) 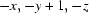; (iii) 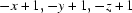; (iv) 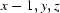; (v) 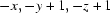; (vi) 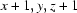; (vii) 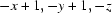; (viii) 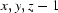; (ix) 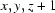; (x) 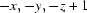; (xi) 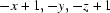.

**Fig. 1.**
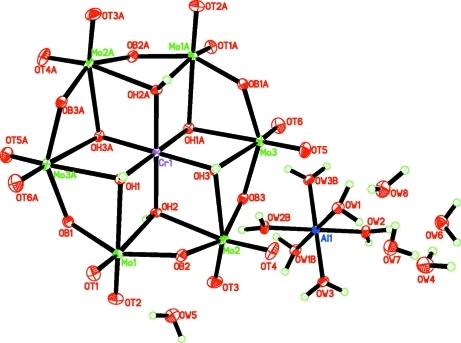
The [Al(H2O)6]3+ cation and the Anderson-type anion as well as the five independent lattice water molecules of the title compound, showing the atom-labelling scheme. Displacement ellipsoids are drawn at the 30% probability level. H atoms have been omitted for clarity. [Symmetry codes: (A) -x, 1 - y, 1 - z; (B)-x, -y, -z.]

**Fig. 2.**
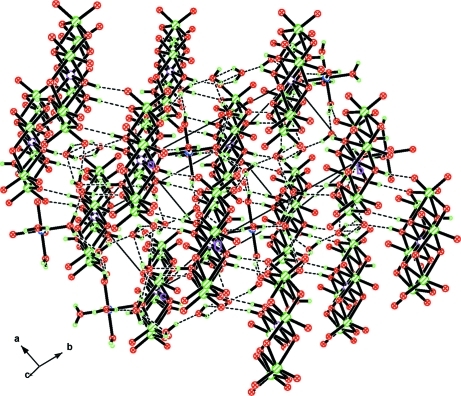
Crystal packing diagram of the title compound, showing the three-dimensional network between the molecular entities through hydrogen bonding (dashed lines).

## supplementary crystallographic information

### Comment

Anderson-type polyoxometalate (POM) anions are frequently used as building blocks to construct extended solid frameworks (An *et al.*, 2005; Shivaiah *et al.*, 2003). As a part of our ongoing study on POMs, we report here the crystal structure of the title compound, [Al(H_2_O)_6_][Cr(OH)_6_Mo_6_O_18_].10H_2_O.

The crystal structure of the title compound (Fig. 1) consists of [Al(H_2_O)_6_]^3+^ cations and Anderson-type [Cr(OH)_6_Mo_6_O_18_]^3-^ anions, both with 1 symmetry. The [Cr(OH)_6_] octahedron lies on the center of six surrounding [MoO_6_] octahedra, all linked through common edges. According to different coordination environments, there are three kinds of O atoms in the anion. The first involves twelve terminal O atoms (labelled OT*x*) that only bind to one Mo atom with distances ranging from 1.691 (4) Å to 1.721 (3) Å. The second type involves six bridging O atoms (OB*x*) shared by two Mo atoms with distances ranging from 1.927 (3) Å to 1.951 (3) Å. The third type involves six O atoms (OH*x*) located between the Mo and Cr atoms with Mo—OH bond lengths ranging from 2.252 (3) Å to 2.314 (3) Å. All these bond lenghths and corresponding angles are in the normal ranges (An *et al.*, 2005).

Abundant hydrogen bonding exist in the title structure (Table 1). The OH groups of the POM anion are involved in forming O—H···O hydrogen bonds to adjacent anions and link the [Cr(OH)_6_Mo_6_O_18_]^3-^ anions into infinite chains along [100]. The chains are further connected with [Al(H_2_O)_6_]^3+^ octahedra through O—H···O hydrogen bonds into sheets lying parallel to (011). The O—H···O hydrogen bonds involving all the lattice water molecules finally link the sheets into a three-dimensional network (Fig. 2).

It should be noted that the title compound is isotypic with its gallium analogue [Ga(H_2_O)_6_][Cr(OH)_6_Mo_6_O_18_].10H_2_O described several years ago (Kaziev, *et al.* 2002). In the latter structure, the Cr^3+^ and Ga^3+^ sites show occupational disorder due to their very similar ionic radius, while such a disorder is not observed in the title compound.

### Experimental

CrCl_3_.6H_2_O (0.532 g, 2 mmol) and Na_2_MoO_4_.2H_2_O (3.629 g, 15 mmol) were dissolved in water (30 ml); ice acetic acid was dropped into the solution until it became clear. The resultant solution was heated for 1 h under stirring and AlCl_3_ (0.267 g, 2 mmol) was added subsequently. The mixture was heated for half an hour and cooled to room temperature. Colorless single crystals were obtained three weeks later after partial evaporation of water.

### Refinement

All H atoms were located from difference Fourier map and treated in the riding mode approximation on their parent atoms, with O—H = 0.85 Å and *U*_iso_(H) = 1.5*U*_eq_(O).

### Crystal data

**Table:**

[Al(H_2_O)_6_][Cr(OH)_6_Mo_6_O_18_]·10H_2_O	*Z* = 1
*M**_r_* = 1332.92	*F*(000) = 647
Triclinic, *P*1	*D*_x_ = 2.630 Mg m^−^^3^
Hall symbol: -P 1	Mo *K*α radiation, λ = 0.71073 Å
*a* = 6.809 (5) Å	Cell parameters from 7543 reflections
*b* = 11.267 (7) Å	θ = 3.2–27.1°
*c* = 11.596 (9) Å	µ = 2.63 mm^−^^1^
α = 101.26 (3)°	*T* = 290 K
β = 97.02 (3)°	Block, colorless
γ = 101.81 (3)°	0.12 × 0.12 × 0.11 mm
*V* = 841.7 (10) Å^3^	

### Data collection

**Table:**

Rigaku R-AXIS RAPID diffractometer	3801 independent reflections
Radiation source: fine-focus sealed tube	3346 reflections with *I* > 2σ(*I*)
graphite	*R*_int_ = 0.037
ω scans	θ_max_ = 27.5°, θ_min_ = 3.2°
Absorption correction: multi-scan (*ABSCOR*; Higashi, 1995)	*h* = −7→8
*T*_min_ = 0.745, *T*_max_ = 0.770	*k* = −14→14
8257 measured reflections	*l* = −15→15

### Refinement

**Table:**

Refinement on *F*^2^	Primary atom site location: structure-invariant direct methods
Least-squares matrix: full	Secondary atom site location: difference Fourier map
*R*[*F*^2^ > 2σ(*F*^2^)] = 0.033	Hydrogen site location: inferred from neighbouring sites
*wR*(*F*^2^) = 0.093	H-atom parameters constrained
*S* = 1.10	*w* = 1/[σ^2^(*F*_o_^2^) + (0.0331*P*)^2^ + 2.719*P*] where *P* = (*F*_o_^2^ + 2*F*_c_^2^)/3
3801 reflections	(Δ/σ)_max_ = 0.009
217 parameters	Δρ_max_ = 0.89 e Å^−^^3^
24 restraints	Δρ_min_ = −0.86 e Å^−^^3^

### Special details

**Table:**

Experimental. (See detailed section in the paper)
Geometry. All e.s.d.'s (except the e.s.d. in the dihedral angle between two l.s. planes) are estimated using the full covariance matrix. The cell e.s.d.'s are taken into account individually in the estimation of e.s.d.'s in distances, angles and torsion angles; correlations between e.s.d.'s in cell parameters are only used when they are defined by crystal symmetry. An approximate (isotropic) treatment of cell e.s.d.'s is used for estimating e.s.d.'s involving l.s. planes.
Refinement. Refinement of *F*^2^ against ALL reflections. The weighted *R*-factor *wR* and goodness of fit *S* are based on *F*^2^, conventional *R*-factors *R* are based on *F*, with *F* set to zero for negative *F*^2^. The threshold expression of *F*^2^ > σ(*F*^2^) is used only for calculating *R*-factors(gt) *etc*. and is not relevant to the choice of reflections for refinement. *R*-factors based on *F*^2^ are statistically about twice as large as those based on *F*, and *R*- factors based on ALL data will be even larger.

### Fractional atomic coordinates and isotropic or equivalent isotropic displacement parameters (Å^2^)

**Table:**

	*x*	*y*	*z*	*U*_iso_*/*U*_eq_	
Al1	0.0000	0.5000	0.5000	0.0162 (4)	
Cr1	0.0000	0.0000	0.0000	0.01424 (19)	
Mo1	0.37029 (5)	0.16663 (3)	−0.10502 (3)	0.01629 (11)	
Mo2	0.33617 (6)	0.24360 (3)	0.17992 (3)	0.01870 (11)	
Mo3	−0.02648 (6)	0.07285 (3)	0.29050 (3)	0.01802 (11)	
OH1	0.2099 (4)	−0.0332 (3)	−0.0983 (3)	0.0156 (6)	
H1	0.2955	−0.0782	−0.0941	0.023*	
OB1	0.1450 (5)	0.1055 (3)	−0.2357 (3)	0.0203 (6)	
OT1	0.5602 (5)	0.1125 (3)	−0.1683 (3)	0.0251 (7)	
OT2	0.4158 (5)	0.3199 (3)	−0.1095 (3)	0.0287 (8)	
OH2	0.1174 (4)	0.1756 (3)	0.0024 (3)	0.0159 (6)	
H2	0.0530	0.2258	−0.0217	0.024*	
OB2	0.4843 (5)	0.1784 (3)	0.0594 (3)	0.0209 (6)	
OT3	0.3656 (6)	0.3945 (3)	0.1671 (3)	0.0321 (8)	
OT4	0.5053 (6)	0.2483 (4)	0.3027 (3)	0.0365 (9)	
OB3	0.0878 (5)	0.2295 (3)	0.2494 (3)	0.0202 (6)	
OH3	0.1830 (5)	0.0372 (3)	0.1527 (3)	0.0166 (6)	
H3	0.2705	−0.0068	0.1480	0.025*	
OT5	0.1586 (6)	0.0662 (3)	0.3999 (3)	0.0315 (8)	
OT6	−0.2117 (6)	0.1163 (4)	0.3617 (3)	0.0334 (8)	
OW1	0.1050 (5)	0.3690 (3)	0.5388 (3)	0.0242 (7)	
H4	0.2073	0.3992	0.5942	0.036*	
H5	0.0411	0.3152	0.5715	0.036*	
OW2	0.0105 (5)	0.5686 (3)	0.6627 (3)	0.0234 (7)	
H6	−0.0143	0.6394	0.6869	0.035*	
H7	0.0689	0.5534	0.7255	0.035*	
OW3	0.2655 (5)	0.5935 (3)	0.5085 (3)	0.0254 (7)	
H8	0.3070	0.6445	0.5758	0.038*	
H9	0.3240	0.6029	0.4491	0.038*	
OW4	0.9017 (6)	0.2342 (3)	0.6599 (4)	0.0379 (9)	
H10	0.9862	0.2141	0.7084	0.057*	
H11	0.8043	0.2487	0.6955	0.057*	
OW5	0.0885 (6)	0.6667 (3)	0.0685 (4)	0.0361 (9)	
H12	0.1879	0.7302	0.0967	0.054*	
H13	0.1180	0.6225	0.0079	0.054*	
OW6	0.1882 (6)	0.5186 (4)	0.8662 (4)	0.0378 (9)	
H14	0.1337	0.4753	0.9112	0.057*	
H15	0.3078	0.5567	0.9018	0.057*	
OW7	0.4894 (4)	0.3814 (3)	0.6484 (3)	0.0359 (9)	
H16	0.5251	0.4239	0.7199	0.054*	
H17	0.4701	0.3052	0.6506	0.054*	
OW8	0.4860 (4)	0.1318 (3)	0.5850 (3)	0.0407 (9)	
H18	0.4573	0.0970	0.6416	0.061*	
H19	0.5475	0.0869	0.5416	0.061*	

### Atomic displacement parameters (Å^2^)

**Table:**

	*U*^11^	*U*^22^	*U*^33^	*U*^12^	*U*^13^	*U*^23^
Al1	0.0168 (9)	0.0139 (8)	0.0166 (9)	0.0037 (6)	0.0007 (7)	0.0011 (7)
Cr1	0.0137 (5)	0.0128 (4)	0.0148 (4)	0.0034 (3)	0.0000 (4)	0.0008 (3)
Mo1	0.01433 (19)	0.01556 (18)	0.0194 (2)	0.00386 (13)	0.00296 (14)	0.00448 (14)
Mo2	0.0168 (2)	0.01554 (18)	0.0188 (2)	0.00120 (13)	−0.00073 (14)	−0.00287 (14)
Mo3	0.0201 (2)	0.01879 (19)	0.01451 (19)	0.00612 (14)	0.00132 (14)	0.00159 (14)
OH1	0.0144 (14)	0.0151 (13)	0.0187 (15)	0.0084 (11)	0.0020 (12)	0.0026 (11)
OB1	0.0223 (16)	0.0205 (14)	0.0188 (16)	0.0051 (12)	0.0015 (13)	0.0073 (12)
OT1	0.0228 (17)	0.0310 (17)	0.0256 (18)	0.0124 (13)	0.0065 (14)	0.0086 (14)
OT2	0.0266 (19)	0.0228 (16)	0.037 (2)	0.0030 (13)	0.0062 (15)	0.0107 (15)
OH2	0.0146 (14)	0.0126 (13)	0.0205 (15)	0.0038 (11)	0.0020 (12)	0.0040 (11)
OB2	0.0183 (16)	0.0221 (15)	0.0199 (16)	0.0041 (12)	0.0001 (13)	0.0017 (12)
OT3	0.036 (2)	0.0178 (15)	0.038 (2)	0.0020 (14)	0.0096 (17)	−0.0011 (14)
OT4	0.030 (2)	0.043 (2)	0.0262 (19)	0.0073 (16)	−0.0073 (16)	−0.0069 (16)
OB3	0.0233 (17)	0.0163 (14)	0.0188 (15)	0.0053 (12)	0.0018 (13)	−0.0007 (12)
OH3	0.0175 (15)	0.0166 (13)	0.0149 (14)	0.0069 (11)	−0.0005 (12)	0.0005 (11)
OT5	0.030 (2)	0.0379 (19)	0.0242 (18)	0.0091 (15)	−0.0051 (15)	0.0070 (15)
OT6	0.036 (2)	0.038 (2)	0.032 (2)	0.0184 (16)	0.0136 (17)	0.0051 (16)
OW1	0.0248 (18)	0.0189 (14)	0.0286 (18)	0.0078 (13)	−0.0001 (14)	0.0047 (13)
OW2	0.0310 (19)	0.0222 (15)	0.0169 (15)	0.0106 (13)	0.0017 (13)	0.0008 (12)
OW3	0.0215 (17)	0.0267 (16)	0.0216 (16)	−0.0024 (13)	0.0032 (13)	−0.0013 (13)
OW4	0.034 (2)	0.0346 (19)	0.050 (2)	0.0118 (16)	−0.0007 (18)	0.0218 (18)
OW5	0.035 (2)	0.0285 (18)	0.044 (2)	0.0106 (15)	0.0019 (18)	0.0068 (16)
OW6	0.036 (2)	0.044 (2)	0.039 (2)	0.0160 (17)	0.0056 (18)	0.0174 (18)
OW7	0.030 (2)	0.048 (2)	0.030 (2)	0.0101 (17)	0.0063 (16)	0.0080 (17)
OW8	0.036 (2)	0.048 (2)	0.035 (2)	0.0049 (18)	−0.0012 (18)	0.0113 (18)

### Geometric parameters (Å, °)

**Table:**

Al1—OW1^i^	1.872 (3)	Mo3—OB1^ii^	1.951 (3)
Al1—OW1	1.872 (3)	Mo3—OB3	1.951 (3)
Al1—OW3^i^	1.877 (3)	Mo3—OH3	2.300 (3)
Al1—OW3	1.877 (3)	Mo3—OH1^ii^	2.330 (3)
Al1—OW2	1.881 (3)	OH1—Mo3^ii^	2.330 (3)
Al1—OW2^i^	1.881 (3)	OH1—H1	0.8500
Cr1—OH3^ii^	1.954 (3)	OB1—Mo3^ii^	1.951 (3)
Cr1—OH3	1.954 (3)	OH2—H2	0.8499
Cr1—OH2	1.970 (3)	OH3—H3	0.8501
Cr1—OH2^ii^	1.970 (3)	OW1—H4	0.8500
Cr1—OH1^ii^	1.983 (3)	OW1—H5	0.8500
Cr1—OH1	1.983 (3)	OW2—H6	0.8501
Mo1—OT2	1.703 (3)	OW2—H7	0.8499
Mo1—OT1	1.721 (3)	OW3—H8	0.8500
Mo1—OB1	1.927 (3)	OW3—H9	0.8500
Mo1—OB2	1.937 (4)	OW4—H10	0.8500
Mo1—OH2	2.252 (3)	OW4—H11	0.8500
Mo1—OH1	2.314 (3)	OW5—H12	0.8559
Mo2—OT4	1.703 (4)	OW5—H13	0.8496
Mo2—OT3	1.709 (4)	OW6—H14	0.8500
Mo2—OB2	1.939 (3)	OW6—H15	0.8500
Mo2—OB3	1.951 (3)	OW7—H16	0.8509
Mo2—OH2	2.283 (3)	OW7—H17	0.8480
Mo2—OH3	2.288 (3)	OW8—H18	0.8499
Mo3—OT6	1.691 (4)	OW8—H19	0.8500
Mo3—OT5	1.699 (4)		
			
OW1^i^—Al1—OW1	180.000 (1)	OT4—Mo2—OH3	94.98 (16)
OW1^i^—Al1—OW3^i^	89.98 (16)	OT3—Mo2—OH3	158.26 (16)
OW1—Al1—OW3^i^	90.02 (16)	OB2—Mo2—OH3	82.20 (13)
OW1^i^—Al1—OW3	90.02 (16)	OB3—Mo2—OH3	71.27 (12)
OW1—Al1—OW3	89.98 (16)	OH2—Mo2—OH3	69.73 (11)
OW3^i^—Al1—OW3	180.0 (2)	OT6—Mo3—OT5	105.4 (2)
OW1^i^—Al1—OW2	89.59 (15)	OT6—Mo3—OB1^ii^	99.75 (17)
OW1—Al1—OW2	90.41 (15)	OT5—Mo3—OB1^ii^	97.91 (16)
OW3^i^—Al1—OW2	90.13 (15)	OT6—Mo3—OB3	99.14 (17)
OW3—Al1—OW2	89.87 (15)	OT5—Mo3—OB3	102.04 (17)
OW1^i^—Al1—OW2^i^	90.41 (15)	OB1^ii^—Mo3—OB3	147.66 (13)
OW1—Al1—OW2^i^	89.59 (15)	OT6—Mo3—OH3	163.68 (15)
OW3^i^—Al1—OW2^i^	89.87 (15)	OT5—Mo3—OH3	89.62 (16)
OW3—Al1—OW2^i^	90.13 (15)	OB1^ii^—Mo3—OH3	84.03 (12)
OW2—Al1—OW2^i^	180.000 (1)	OB3—Mo3—OH3	71.00 (12)
OH3^ii^—Cr1—OH3	180.00 (18)	OT6—Mo3—OH1^ii^	95.90 (16)
OH3^ii^—Cr1—OH2	96.53 (13)	OT5—Mo3—OH1^ii^	157.46 (14)
OH3—Cr1—OH2	83.47 (13)	OB1^ii^—Mo3—OH1^ii^	70.90 (12)
OH3^ii^—Cr1—OH2^ii^	83.47 (13)	OB3—Mo3—OH1^ii^	81.26 (13)
OH3—Cr1—OH2^ii^	96.53 (13)	OH3—Mo3—OH1^ii^	70.18 (12)
OH2—Cr1—OH2^ii^	180.00 (18)	Cr1—OH1—Mo1	101.22 (12)
OH3^ii^—Cr1—OH1^ii^	94.97 (14)	Cr1—OH1—Mo3^ii^	101.37 (13)
OH3—Cr1—OH1^ii^	85.03 (14)	Mo1—OH1—Mo3^ii^	92.79 (11)
OH2—Cr1—OH1^ii^	95.90 (12)	Cr1—OH1—H1	131.8
OH2^ii^—Cr1—OH1^ii^	84.10 (12)	Mo1—OH1—H1	110.7
OH3^ii^—Cr1—OH1	85.03 (14)	Mo3^ii^—OH1—H1	111.9
OH3—Cr1—OH1	94.97 (14)	Mo1—OB1—Mo3^ii^	120.22 (16)
OH2—Cr1—OH1	84.10 (12)	Cr1—OH2—Mo1	103.79 (12)
OH2^ii^—Cr1—OH1	95.90 (12)	Cr1—OH2—Mo2	103.22 (13)
OH1^ii^—Cr1—OH1	180.00 (15)	Mo1—OH2—Mo2	93.30 (12)
OT2—Mo1—OT1	104.94 (17)	Cr1—OH2—H2	125.8
OT2—Mo1—OB1	97.87 (16)	Mo1—OH2—H2	106.7
OT1—Mo1—OB1	101.08 (16)	Mo2—OH2—H2	118.4
OT2—Mo1—OB2	100.85 (16)	Mo1—OB2—Mo2	116.61 (16)
OT1—Mo1—OB2	96.87 (16)	Mo2—OB3—Mo3	118.42 (15)
OB1—Mo1—OB2	149.69 (14)	Cr1—OH3—Mo2	103.55 (13)
OT2—Mo1—OH2	94.45 (14)	Cr1—OH3—Mo3	103.34 (14)
OT1—Mo1—OH2	159.55 (14)	Mo2—OH3—Mo3	93.88 (11)
OB1—Mo1—OH2	82.26 (14)	Cr1—OH3—H3	110.0
OB2—Mo1—OH2	72.74 (13)	Mo2—OH3—H3	110.7
OT2—Mo1—OH1	162.67 (14)	Mo3—OH3—H3	131.6
OT1—Mo1—OH1	90.85 (14)	Al1—OW1—H4	108.5
OB1—Mo1—OH1	71.64 (12)	Al1—OW1—H5	122.9
OB2—Mo1—OH1	83.95 (13)	H4—OW1—H5	97.7
OH2—Mo1—OH1	70.89 (11)	Al1—OW2—H6	122.0
OT4—Mo2—OT3	105.5 (2)	Al1—OW2—H7	131.7
OT4—Mo2—OB2	98.10 (17)	H6—OW2—H7	103.9
OT3—Mo2—OB2	101.59 (16)	Al1—OW3—H8	111.0
OT4—Mo2—OB3	99.29 (18)	Al1—OW3—H9	125.4
OT3—Mo2—OB3	97.85 (16)	H8—OW3—H9	120.8
OB2—Mo2—OB3	149.30 (13)	H10—OW4—H11	107.7
OT4—Mo2—OH2	162.45 (15)	H12—OW5—H13	108.5
OT3—Mo2—OH2	90.88 (16)	H14—OW6—H15	107.7
OB2—Mo2—OH2	72.01 (13)	H16—OW7—H17	107.5
OB3—Mo2—OH2	84.24 (13)	H18—OW8—H19	107.7

Symmetry codes: (i) −*x*, −*y*+1, −*z*+1; (ii) −*x*, −*y*, −*z*.

### Hydrogen-bond geometry (Å, °)

**Table:**

*D*—H···*A*	*D*—H	H···*A*	*D*···*A*	*D*—H···*A*
OH3—H3···OT1^iii^	0.85	1.85	2.681 (4)	167.
OH1—H1···OB2^iii^	0.85	2.10	2.944 (4)	172.
OH2—H2···OW5^iv^	0.85	1.82	2.665 (5)	178.
OW3—H8···OT4^v^	0.85	1.83	2.628 (5)	156.
OW3—H9···OW7^v^	0.85	1.81	2.632 (5)	163.
OW1—H4···OW7	0.85	2.01	2.730 (5)	142.
OW1—H5···OW4^vi^	0.85	1.74	2.577 (5)	167.
OW2—H6···OB3^i^	0.85	1.72	2.559 (4)	171.
OW2—H7···OW6	0.85	1.88	2.728 (5)	178.
OW4—H10···OB1^vii^	0.85	1.94	2.737 (5)	156.
OW5—H12···OT1^viii^	0.86	2.15	3.002 (6)	179.
OW5—H13···OW6^ix^	0.85	1.99	2.842 (6)	180.
OW6—H14···OW5^i^	0.85	2.04	2.800 (6)	149.
OW6—H15···OT3^v^	0.85	2.45	3.091 (6)	133.
OW7—H16···OT3^v^	0.85	2.13	2.878 (5)	146.
OW7—H17···OW8	0.85	1.98	2.759 (5)	152.
OW8—H18···OT1^x^	0.85	2.19	2.902 (5)	141.
OW8—H18···OT6^xi^	0.85	2.62	3.241 (5)	131.
OW8—H19···OW8^xii^	0.85	2.56	3.283 (6)	144.

Symmetry codes: (iii) −*x*+1, −*y*, −*z*; (iv) −*x*, −*y*+1, −*z*; (v) −*x*+1, −*y*+1, −*z*+1; (vi) *x*−1, *y*, *z*; (i) −*x*, −*y*+1, −*z*+1; (vii) *x*+1, *y*, *z*+1; (viii) −*x*+1, −*y*+1, −*z*; (ix) *x*, *y*, *z*−1; (x) *x*, *y*, *z*+1; (xi) −*x*, −*y*, −*z*+1; (xii) −*x*+1, −*y*, −*z*+1.
